# Body Image in Borderline Personality Disorder: A Systematic Review of the Emerging Empirical Literature

**DOI:** 10.3390/jcm10184264

**Published:** 2021-09-20

**Authors:** Magdalena Wayda-Zalewska, Barbara Kostecka, Katarzyna Kucharska

**Affiliations:** 1 Institute of Psychology, Cardinal Stefan Wyszynski University in Warsaw, 01-938 Warsaw, Poland; m.waydazalewska@student.uksw.edu.pl (M.W.-Z.); k.kucharska@uksw.edu.pl (K.K.); 2 II Department of Psychiatry, Medical University of Warsaw, 03-242 Warsaw, Poland

**Keywords:** borderline personality disorder, body image, body perception, body dissatisfaction

## Abstract

As an element of distorted self-image, body image disturbances may be relevant to borderline personality disorder (BPD). Therefore, this systematic review aims to critically discuss and summarize empirical findings in this matter. Based on the available theoretical models, three body image components were identified: (a) perception, (b) affect and cognition, and (c) general body dissatisfaction. We conducted a systematic search of the empirical literature published in English in the MEDLINE, PsycINFO, and Scopus databases until June 2021 using a priori eligibility criteria (BPD; BPD symptoms or features in nonclinical groups; quasipsychotic or psychotic symptoms were not considered). We included *k* = 10 records meeting the criteria. Compared with other analyzed groups, individuals diagnosed with BPD obtained higher scores in the three components of body image disturbances. The issue of body image in BPD is relatively understudied, although current research findings clearly indicate disturbances in all of the abovementioned body image components in individuals with BPD or significant relationships of these components with BPD traits or symptoms both in clinical and nonclinical samples. Eventually, possible practical implications and future research directions are also discussed.

## 1. Introduction

Borderline personality disorder (BPD) is a complex, heterogeneous, and severe mental condition characterized by persistent substantial instability of emotions, interpersonal relationships, and self-image. Whereas the first two functional domains of BPD have been frequently addressed in the empirical literature, the distorted self-image has received relatively little attention despite potentially playing a key role in the development, maintenance, therapy, and treatment of this disorder [1]. There is an urgent need to investigate the relationship between BPD and distorted body image, as it may be of great clinical importance in the treatment of BPD. Some researchers have already acknowledged the relevance of body image in BPD and suggested exploring whether distorted body image in BPD has an impact on self-harm behaviors and quasipsychotic states such as dissociation or derealization [2,3]. 

The definition of body image is not simply restricted to the physical aspects of the body. In some theoretical frameworks, body image is conceptualized as: (a) a body schema and (b) an emotional relationship that a person has with their own body [4]. According to those frameworks, a body schema is a cognitive-informative aspect of the body—it relates to the overall “body of knowledge” that one has about their body (regardless of emotional involvement). An emotional relationship, as opposed to the body schema, is a more complex construct. It consists of behavioral, emotional, and cognitive components and refers to the level of satisfaction with one’s own body and emotional responses elicited by a particular level of satisfaction. Body image satisfaction is the function of distance between the perceived body image and the ideal body image at any time. Thus, in this conceptualization, the level of body image satisfaction develops through a dynamic process that changes over time and depends on the perceived and ideal body images. When it comes to mental disorders, disturbed body image is typically investigated in relation to eating disorders (see, e.g., [5]). Even though it is justified by the *Diagnostic and Statistical Manual of Mental Disorders, Fifth Edition* (*DSM-5*) [1] and *International Classification of Diseases, Tenth Revision* (*ICD-10*) [6] diagnostic criteria, eating disorders are not the only group of mental disorders in which body image may be disturbed. BPD is often comorbid with eating disorders [7] and these diagnostic entities share several characteristics, e.g., self-destructive behaviors (binge eating and purging in bulimia nervosa, food restriction in anorexia nervosa, and self-injury in BPD) or difficulties in regulating emotions (see [1]). However, it seems that body image in individuals with BPD may be disturbed not only due to comorbid eating disorders but also due to BPD alone. First of all, patients with BPD struggle with identity disturbance that encompasses, e.g., difficulties with self-acceptance as well as lack of stability and integrity of self-image or sense of self. 

Self-image is defined as a collection of perceptions of the self that include: an image of the body, impressions of one’s capabilities and personality [8] or mental picture that includes physical appearance, and the integration of desires, experiences, and feelings [9]. Regardless of the definition of self-image adopted, there is no doubt that body image constitutes its central component. According to Kernberg (1978), patients with BPD experience the so-called splits in their affect and thinking, known as identity diffusion, which manifests with contradictory character traits, discontinuity of the self, and either very idealized or devalued object relations. Both identity diffusion and identity distress were significant predictors of appearance evaluation and body satisfaction [10]. Moreover, patients with BPD often have negative attitudes towards their bodies. They tend to have lower self-esteem than those with social phobia [11]. In BPD self-esteem is significantly related to body image, because patients tend to evaluate their own physical attractiveness as extremely low and declare low satisfaction and trust in relation to their body image, as well as high levels of discomfort in that regard [2]. In addition, they are inclined to believe that physical attractiveness is an important factor in the pursuit of happiness. Apart from that, the subjective perception of physical attractiveness in patients with BPD has been related to avoidance behaviors, which constitute BPD symptoms [12]. 

The aim of this systematic review is to analyze, summarize, and synthesize all the available results of empirical studies on body image in patients with BPD or the relationship between body image and BPD traits or symptoms in nonclinical samples. To the best of our knowledge, this is the first peer-reviewed systematic review of studies on body image in BPD. Another aim of this review is to identify and discuss constructs of body image based on our findings. Finally, we outline future directions and therapeutic implications pertaining to body image disturbances.

## 2. Methods

### 2.1. Search Strategy

**Figure 1:**
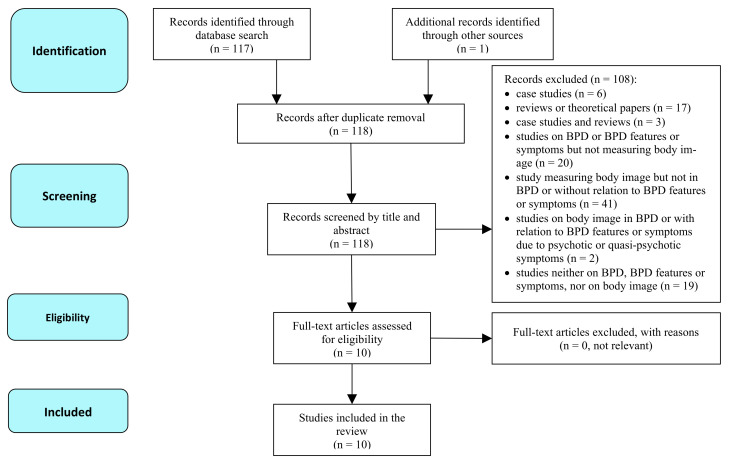
PRISMA flow diagram of the search strategy.

A comprehensive search of the MEDLINE, PsycINFO, and Scopus databases for articles published until 1 June 2021, was conducted based on the PRISMA guidelines [13] (see Figure 1). The following search algorithm was used: (“borderline” OR “BPD”) AND (“body image” OR “body regard” OR “body perception” OR “body dissatisfaction” OR “body satisfaction” OR “body attractiveness” OR “body unattractiveness” OR “body self-evaluation” OR “body evaluation”). Duplicated articles were automatically removed by the search engines. In addition, publications citing the included articles were checked via Scopus, and reference lists of the reviewed articles were hand-searched. The articles selection and assessment was done by all authors, and the search was done by two researchers independently.

### 2.2. Inclusion Criteria

Articles were included in our review if they met the following criteria: written in English; published in peer-reviewed scientific journals; discussed original empirical studies on body image; involved: (1) patients with BPD or (2) analysis of BPD symptoms or features in nonclinical groups; did not concern body perception distortions resulting from quasipsychotic (i.e., depersonalization or derealization) or psychotic symptoms (excluded due to the temporal nature of those states).

## 3. Results

### 3.1. Characteristics of the Studies

The search finally yielded ten articles published between 1992 and 2021 (see Table 1). Only four studies involved patients with BPD without any specific comorbid mental disorder (see [12,14,15,16]). Two of them [14,15] also compared BPD with another mental disorder (ie, post-traumatic stress disorder [PTSD]). A single study involved only patients with BPD and another comorbid condition (i.e., PTSD) (see [17]). Two studies included clinical samples without BPD (see [2,18] and other two studies involved nonclinical samples without BPD (see [19,20]). In one study, BPD features were analyzed in high-school girls with no documented diagnosis of BPD (see [21]). Almost all studies involved only female participants (for a mixed sample, see [20]) and used cross-sectional self-report scales (for an experiment, see [17]; for cross-sectional visual evaluations, see [15,16]). Six studies were conducted in Germany (see [12,14,15,16,17,19] and four studies were carried out in English-speaking countries (see [2,18,20,21]).

The studies examined various dimensions of body image and employed different measures to investigate it (see Table 2). Three of the studies addressed the issue of body perception [18,19,20]. Four papers investigated affect and cognition [2,12,14,17]. Three studies concerned global body dissatisfaction [15,16,21]. One study was experimental and used Emotional Stroop Task with body-related words to assess biased information processing [17]. All but one mentioned study were cross-sectional and employed self-report measures of body image.

### 3.2. Results by Body Image Components

#### 3.2.1. Body Perception (Body Regard, Body Attitude)

Sansone et al. [18] used the Eating Disorders Inventory (EDI; [25]), which is a self-reported scale of eating-related attitudes and traits. Participants also completed the Body Image Avoidance Questionnaire (BIAQ; [23]). The study reported that Personality Diagnostic Questionnaire—Revised (PDQ-R) scores moderately positively correlated with body mass index (BMI). Also, there were significant relationships between the severity of BPD symptoms and measures of body image perception, such as general body dissatisfaction, self-related bodily attractiveness, self-related facial attractiveness, and self-avoidance due to body image concerns. Moreover, overweight, and obese women had significantly higher scores on the PDQ-R scale compared with other participants, which suggested a relationship between BPD and obesity. 

Muehlenkamp et al. [20] hypothesized that body regard may be a moderator in the association between emotional dysregulation in BPD and non-suicidal self-injury (NSSI). To measure body image perception, four subscales from the Body Attitudes Scale (BAS; [22]) were used, i.e., body integrity, attractiveness, health, and effectiveness; with higher scores indicating more positive body attitudes or body regard. Those subscales have been used in previous research on body attitudes among adolescents who engaged in self-injury. BPD symptoms and negative affect were associated with NSSI. Negative associations between body regard and negative affect, emotional dysregulation, and BPD symptoms were also observed. There was also a positive association between NSSI and emotional dysregulation, BPD symptoms, and negative affect. The frequency of NSSI was significantly associated with emotional dysregulation only in the case of low body regard. No significant association was found among individuals with moderate or high body regard.

Dyer et al. [19] combined two questionnaires: Multidimensional Body–Self Relations Questionnaire—Appearance Scales (MBSRQ-AS; [28,29]) and Fragebogen zur Erfassung des Körperbildes nach Brandverletzungen [FKBB; English: Evaluation of Body Image After Burn Injuries; [27]). MBSRQ-AS assess self-attitudinal aspects of body image (including affective, cognitive, and behavioral components) with 34 question items, while FKBB focuses on specific issues relevant for individuals with scars. Correlations between BMI and BPD symptoms as well as size and appearance of scars were found. The NSSI group exhibited significantly higher scores with respect to typical symptoms of BPD, frequency of self-harm behaviors, and perception of scar appearance compared with the group with scars after an accident or surgery. The NSSI groups differed with regard to three out of five subscales of the body image components assessed by MBSRQ-AS: appearance evaluation, body area satisfaction scale, and overweight preoccupation.

#### 3.2.2. Affect and Cognition 

MBSRQ-AS was used by Dyer et al. [12] to assess aspects of body image, which include affective, cognitive, and behavioral components (five subscales: Appearance Evaluation, Appearance Orientation, Body Areas Satisfaction, Overweight Preoccupation, and Self-Classified Weight). The Body Image Avoidance Questionnaire (BIAQ) was used to evaluate avoidance behavior with respect to clothing, social activities, eating restraints, and weight. The results indicated that patients with BPD significantly differed from healthy controls (HCs) in terms of the mean BIAQ score and MBRSQ scales (except the Appearance Orientation subscale). It was concluded that they had more disturbed body image than HCs. Another between-group analysis showed that BPD patients with life diagnosis of eating disorders (LDED) had more concerns with regard to eating, weight, body shape, and restraints to their bodies (Eating Disorder Examination Questionnaire [EDE-Q] subscales); had higher scores on the MBSRQ Overweight Preoccupation subscale and a higher mean BIAQ score. BPD patients with a history of child sexual abuse (CSA) compared with BPD patients without CSA reported more frequent avoidance behavior in relation to their body (higher mean BIAQ scores) and greater dissatisfaction with their overall appearance (MBSRQ Appearance Evaluation subscale) and different areas of their body (MBSRQ Body Areas Satisfaction subscale).

The Emotional Stroop Test (EST) was performed to assess biased information processing in a group of patients with PTSD (caused by CSA; with or without diagnosed BPD) and HCs in a study by Witthöft et al. [17]. Results indicated that patients with PTSD and BPD showed a stronger attentional bias to body-related words compared with the HC group and a marginally stronger emotional interference effect compared with the PTSD patients without BPD. 

Three subscales (Body Image Vulnerability Scale, Self-Investment Scale, Appearance Stereotyping Scale) of the Appearance Schemas Inventory—Revised (ASI-R) were used in a study by Sansone et al. [2] to measure the extent to which individuals hold core beliefs about the importance, meaning, and effects of appearance in their own and other people’s lives. Participants who scored positively (exceeded the clinical cutoff) on either or both BPD (PDQ) and self-harm scales (SHI) were more likely to report body image vulnerability, appearance stereotyping, low private self-consciousness, low public self-consciousness, and a lack of familiarity with one’s own body. 

In a study by Dyer et al. [14], a modified Survey of Body Areas (SBA; [15]) was used to investigate attitudes toward 26 different body areas in two patient groups. Different body areas were marked in drawings (breasts, hips, hair, pubic area), and participants were asked to rate emotions they experienced (interest, happiness, pride) when looking at specific areas of their own body. Additionally, participants indicated if a particular area was associated with a traumatic event. Significant correlations were found in the PTSD + BPD group regarding “Mean rating of body-related emotions.” All patient groups rated body-related emotions more negatively than HCs, whereas patients diagnosed with PTSD (with comorbid BPD) reported more negative feelings related to their body as compared with BPD patients without PTSD. Patients diagnosed with PTSD (with and without BPD) reported more trauma-associated body areas than HCs and BPD patients without comorbid PTSD. Shame and disgust correlated with anger expression but were not associated with anger as a trait (STAXI-TA).

#### 3.2.3. General Body Dissatisfaction

To compare general body dissatisfaction, the 8-item Body Dissatisfaction Scale (BDS; [25]) was used in the study by Steiger et al. [21], which measures attitudes toward different body parts. Participants were divided into four groups: BD/B (body dissatisfaction + BPD traits—high risk group), BD (only body dissatisfaction), BORD (BPD only), and NR (not-at-risk participants). There was also a group of patients diagnosed with bulimia nervosa (BN). Participants with BD/B had an elevated profile of disordered eating and associated disturbances highly similar to those in the BN group. Post-hoc test comparison scores showed that the level of body dissatisfaction did not differ among the high-risk (BD/B), BD, and BN groups. Similarly, mean Borderline Syndrome Index (BSI; [30]) scores in the groups presenting borderline features (BD/B and BORD) and in the BN group were higher than mean scores of the participants without BPD traits (BD and NR).

Kleindienst et al. [15] used the Survey of Body Areas (SBA; [14]; original version: [15]) to indicate which body areas participants liked or disliked and to mark the sites where physical scars were located. Patients with BPD showed different patterns and general evaluation of their own body compared with other groups, but the negative evaluation of their own body was similar to that observed in the group of patients with PTSD after CSA. The mean score of evaluation of body areas varied across the four analyzed groups (*p* < 0.0001). The average body rating in HCs was higher than the neutral value in contrast to patients with BPD whose ratings were clearly negative. Ratings in the clinical control groups were in the middle of the scale in between: they were negative in the PTSD group, but no clear trend was observed for patients with other anxiety disorders. In a subgroup analysis, patients with BPD and CSA and those with BPD and without CSA negatively rated body areas with at least one scar as well as body areas unaffected by scars, so the results were very similar.

The authors of that study also compared the key indicators of the SBA and MBSRQ scales. These strongly correlated with body image in terms of the number of both positively and negatively assessed areas of appearance. 

SBA was also used in another study by Kleindienst et al. [16]. Participants were asked to color the body areas they like or dislike. Mean ratings of body evaluation and separate mean values for sexual and neutral areas were calculated. The mean evaluation score for body areas differed across the three study groups: the most positive score was reported in HCs, a predominantly positive one in a group of patients after BPD remission, and an overall negative one in patients with active BPD. The evaluation of body areas that typically have a sexual connotation was positive in HCs and negative in those after BPD remission and patients diagnosed with active BPD.

## 4. Discussion

This systematic review outlined findings from reports on differences in body image disturbances among individuals diagnosed with BPD, other clinical groups, and gender-matched controls. To the best of our knowledge, this is the first peer-reviewed systematic review of data on body image in BPD. We categorized the aspects of body image disturbances covered in the reviewed papers into three areas: a. perception, b. affect and cognition, and c. general body dissatisfaction. 

Findings from the studies reviewed consistently indicate disturbances in all the above- mentioned body image components in BPD or significant relationships of those disturbances with BPD traits or symptoms. Therefore, our conclusions are in line with results from previous studies regarding the relationship between BPD and distorted body image. Our findings are also consistent with those obtained in studies on BPD with comorbid eating disorders or eating disorder symptoms, which indicates stronger body image disturbances in these groups in the following components: perceptive, subjective satisfactory, affective/cognitive, and behavioral. 

The reviewed articles suggest that the experience of CSA or PTSD after CSA is associated with a more negative body image in patients with BPD [12,14,15,16,17], but some negative emotions such as shame, guilt, anger, and disgust are more markedly associated with PTSD syndrome and CSA than BPD symptoms [14]. The results also suggest that specific areas (pubic area, buttocks, inner thighs) are associated with trauma and highly aversive emotions. Thus, the patient’s body may act as a trigger for traumatic memories see [14]. In addition, the degree of body image disruption appears to correlate with the severity of experienced violence [31]. While some studies suggest that CSA is an aggravating factor for the negative evaluation of one’s own body [12], some other results show that experience of CSA and/or PTSD may be unrelated to body self-evaluation in BPD see [15]. As mentioned earlier, patients diagnosed with BPD but without PTSD (after CSA) report higher level of disgust than healthy individuals yet show lower level of disgust than patients with PTSD see [14]. In some studies, i.e., [11], patients with BPD (with or without PTSD) had higher level of body disgust compared with HCs, but there were no significant differences between the two groups. Therefore, the influence of PTSD on the sense of body disgust in BPD requires further investigation. More severe body image abnormalities may be due to post-CSA PTSD or more severe BPD symptoms, such as emotional dysregulation, which is characteristic for patients with BPD and may, in combination with PTSD, be necessary to produce a stronger attention focus on body-related stimuli and body-related bias. Further empirical studies are needed to assess whether negative body evaluation in patients with BPD affects all body areas or is pronounced for areas that are typically affected by CSA (i.e., sexually connoted). Additionally, the negative evaluation of sexually connoted body areas seems to remain an issue even after disorder remission has been achieved [18], which is a specific therapeutic challenge and requires a systematic evaluation of treatment modules.

Research suggests that negative body regard may be the factor that differentiates the groups that engage in self-harm behavior from those who do not. Up to 90% of patients with BPD engage in repetitive NSSI behaviors and, on average, 20.4% of patients with BPD have at least one scar in 43 areas of the body (see [15]). One of the reviewed studies [19] indicated that patients with BPD symptoms who engaged in NSSI reported a more negative body image compared with those with scars of another origin. The negative evaluation of one’s own appearance might aggravate NSSI, as there is a proven relationship among emotional dysregulation, NSSI, and low body regard [20]. Individuals may vary in terms of risk for NSSI depending on their body image. Some patients admit that they sometimes harm themselves against their own will and that they feel negative emotions toward themselves, such as shame, guilt, disgust, or self-criticism through self-punishment. 

As concluded in previous studies, self-inflicted scars seem to have a great influence on distorted body image and promote negative body evaluation, as they may recall traumatic experiences, trigger stressful memories, and lead to body image distress [15,19,31]. However, some patients feel proud of their scars, as evidence about what they have been through. This relationship between scars and the negative evaluation of the affected areas seems specific to patients with BPD [15]. Scars from self-inflicted injuries can very likely influence body image concerns, but non–self-inflicted scars may also have a negative impact on body image [19]. Therefore, the influence of self-inflicted scars on body evaluation in BPD requires further elaboration.

Although there is a confirmed relationship between BMI and BPD symptoms, severe body image disturbances appear in individuals with BPD regardless of their weight status [18]. When it comes to the lifetime diagnosis of a comorbid eating disorder, it may aggravate body image disturbances in BPD with respect to avoidance behavior and concerns related to body weight and shape. Some other body image disturbances, e.g., regarding satisfaction with one’s own body and appearance, can also be identified, independently from a lifetime diagnosis of a comorbid eating disorder [12].

Further study results [2] have shown that several body image measures, such as defects in appearance and social unacceptability, assumptions about social goodness or badness as a function of an attractive or unattractive appearance, and degree to which an individual focuses attention on those aspects of the self that are or are not observable to others, seem to have significant associations with BPD. Nearly all these variables appear to subtly and predominantly reflect cognitive processes. 

As a conclusion, BPD seems to be strongly associated with an unstable, disturbed self-image. People with BPD struggle with fragile self-esteem, insecure self-image, and dependence on others, as well as the influence of external factors. This may create a propensity toward substance abuse and may be shifted to eating disturbances in an attempt to regulate mood, modulate stress, and maintain a sense of personal integrity [1]. Finally, a wide range of mood- and impulse-regulation problems in BPD influence self-image and body image. Individuals diagnosed with BPD evaluate their own appearance more negatively than their peers without BPD and are more likely to believe that attractiveness is an important factor for happiness and acceptance. Many of these variables may be shaped by perception processes, which likely account for more severe cognitive distortions seen in individuals with BPD. Our review identified various components of distorted body image in BPD, which related to body perception, cognitive-affective body image representation, and general body dissatisfaction. These components seem to be closely related to BPD symptoms described in DSM-5 [1] (such as NSSI and emotional dysregulation) as well as to comorbid eating disorders, PTSD, and a history of CSA. Presumably, interventions directed towards improving body image may result in decreased rates of self-destructive behaviors and there is a need to integrate this issue into the therapeutic process. What is more, some prospective studies have suggested that such interventions may contribute to the reduction of the suicide risk [32,33]. 

### 4.1. Limitations

This systematic review is based on comprehensive searches using three multidisciplinary databases and on an additional hand search of reference lists of the relevant articles. The search algorithm included 11 different terms frequently used as keywords or part of the titles of the articles related to the issue of body image in/and BPD. However, this paper included only articles published in English, therefore, we may not have identified some relevant studies written in other languages. In addition, we did not include a meta-analysis in our systematic review; however, it would be of limited usefulness due to different body image components covered across studies.

The important limitation seems to be the fact that the number of studies on body image in BPD is still far too small. That may be the result of the fact that there is no direct reference to body image in the diagnostic criteria of BPD in DSM-5 or in its theoretical conceptualizations, and until now, the subject of body image has been undertaken in empirical research mainly in the area of eating disorders or dysmorphophobia. So far, this issue has been addressed only by a few research teams, mainly from Western Europe and North America, which may reduce the possibility of generalizing the conclusions to other regions of the world. Further, the most frequent methodological limitation of the available studies is the fact that they report on only female samples and use exclusively self-report scales as body image measures. Also, some of the studies reviewed did not contain information about mean BMI (which could be relevant for body image research) or reported significant between-group age differences. Hence, the influence of those factors on findings cannot be excluded.

### 4.2. Future Directions and Practical Clinical Implications

To increase the representativeness of the data, an important future direction is to expand the number of empirical studies concerning body image in BPD, especially in underrepresented regions of the world, such as Latin America, Eastern Europe, Asia or Africa.

When it comes to methodological suggestions, future research should focus on individuals with BPD, especially on those from clinical samples, including adolescents, to capture the developmental trajectories of distorted body image and to better adjust therapeutic methods to the needs of such individuals. This should also be studied in men with BPD, taking into account gender differences in body appreciation in the general population (for a meta-analysis, see [34]). Body image in BPD needs further investigation also in comparison to other mental disorders in which it seems distorted, such as anorexia nervosa, bulimia nervosa, or body dysmorphic disorder (cf. [35]), so that its specific and transdiagnostic aspects could be captured and appropriately tailored in therapeutic interventions.

Furthermore, future studies should also investigate body image in BPD with the use of other self-report scales (for a review, see [26]) to validate the currently available findings. The experience sampling method (ESM) should be implemented in clinical research to capture the daily dynamics of body image and to identify factors contributing to these fluctuations, such as the interaction between the situational context (e.g., hearing negative comments on one’s body from someone else) and related emotions (cf. [36]). In addition, functional magnetic resonance imaging (fMRI) could be used to identify the underlying neural mechanisms of disturbed body image in BPD (cf. [37,38]).

Furthermore, it would be worth investigating whether body image is also abnormal in BPD individuals without a history of sexual abuse in childhood or adolescence. Although most individuals with BPD or with severe BPD symptoms or traits may have substantial body image disturbances, future research should compare subgroups of such individuals who have such difficulties with those who do not and try to identify factors that may account for this difference, taking into account the findings of Steiger et al. see [21].

In terms of possibly relevant constructs, future studies should explore the link between body image disturbances and body modifications in BPD, such as piercing, tattooing, scarifications (cf. [39]), or plastic surgeries (cf. [40]). Patients undergoing aesthetic medicine procedures have higher indices of body image disturbances and show a higher prevalence of comorbid body dysmorphic disorder (BDD) [41] and personality disorders (PDs) or PD traits than controls [42]. Some studies suggest that the presence of PDs may not have a direct influence on the choice of aesthetic plastic surgery, while many studies highlight that an abnormal personality profile (with greater frequency of cluster B PDs, e.g., narcissistic PD, histrionic PD, and BPD) represent one of the predisposing factors for the development of distortions of body image and undergoing plastic surgery. Thus, the diagnostic process in individuals with BPD appears essential in the search for other psychiatric comorbidities to better understand the nature of body image disturbances. If the mechanisms are better understood, therapeutic interventions might be improved to prevent making serious decisions on plastic surgery, which individuals with BPD can regret later, because such body modifications may become more ego-dystonic as the therapeutic process progresses. Surprisingly, such basic constructs for body image as general self-esteem, self-disgust or shame have not been included in research on this topic so far, although they seem to play a crucial role in body image disturbance (see, e.g., [43,44]).

One of the evidence-based approaches in body image psychotherapy is cognitive-behavioral therapy (CBT). This treatment method offers various techniques and interventions that help reduce cognitive and perceptual disturbances regarding one’s body image, such as selective perception [45]. Other evidence-based psychotherapeutic approaches include: dialectical behavior therapy (DBT), schema-focused therapy (SFT), or transference-focused therapy (TFT) [46]. DBT, the most evidence-based approach, may be particularly effective in promoting the positive view of the body and improvement of body image in BPD. The influence of DBT on body image therapy is multidimensional, which has been also confirmed in numerous studies. Some more recent therapeutic interventions targeted at body image disturbances include evaluative conditioning (cf. [47]) or mirror exposure therapy [5]. However, further research is necessary to explore the efficacy of those therapies in patients with BPD.

## Figures and Tables

**Table 1:** Characteristics of the reviewed studies.

Study	Country	Group (Overall Sample Size; no. of Women); Clinical Sample Type	Mean Age (SD), Age Range [Years]	Mean Education Level [Years of Education or Degree]	Mean BMI (SD), BMI Range	Study Design	BPDMeasure	Body Image Measure	Main Results	Results by Components of Body Image Disturbances	Limitations
Dyer, Borgmann, et al. [12]	Germany	BPD (N = 89; 89); HCs (N = 41; 41); inpatients and outpatients	MBPD = 31.09 (9.15), NI; MHCs = 31.57 (11.53), NI	NI	MBPD = 26.97 (7.64), NI; MHCs = 25.99 (5.79), NI	Cross-sectional	IPDE, BSL-23	BIAQ, MBSRQ-AS	History of CSA and comorbid ED independently related to ↑ negative body image	Affect/Cognition: BPD > HC in mean BIAQ and MBSRQ; BPD > HC in BID; BPD + LDED > BPD -LDED in BID	Only self-reported measures of body image; no mean education level; only women
Dyer, Feldmann, et al. [18]	Germany	BPD (N = 25; 25); PTSD (N = 23; 23); PTSD + BPD (N = 22; 22); HCs (N = 27; 27); inpatients and outpatients	MBPD = 31.44 (9.31), NI MBPD + PTSD = 30.57 (9.08), NI; MPTSD = 38.91 (8.59), NI; MHCs = 30.82 (10.33), NI	NI	NI, NI	Cross-sectional	IPDE	Modified SBA	↑ negative emotions toward the body in patients with BPD than in HCs, but ↓ negative than in patients with BPD + PTSD and those with PTSD	Affect/Cognition: BPD + PTSD > BPD -PTSD in negative feelings related to their body;	Significant age differences between the BPD and PTSD groups; no mean education level; no mean BMI; only women; small sample sizes
Dyer, Hennrich, et al. [22]	Germany	NSSI (N = 56; 56); A/S (N = 69; 69); general	MNSSI = 27.39 (8.42); MA/S = 27.23 (10.18); NI for each group separately, but 18–60 for the whole sample	NI	MNSSI = 25.57 (8.29), NI; MA/S = 23.47 (6.06), NI	Cross-sectional	BSL-23	FKBB (EBIABI), MBSRQ-AS	↑ negative body image in individuals engaged in NSSI than in individuals with A/S	Perception: NSSI > A/S in BPD symptoms, quantity of self-harming behaviors, perception of appearance of the scar; NSSI > A/S in 3/5 assessed BID components	Only self-reported measures of BPD and body image; no mean education level; only women
Kleindienst et al. [20]	Germany	BPD (N = 26; 26); rBPD (N = 22; 22); HCs (N = 20; 20); inpatients and outpatients	MBPD = 31.65 (9.09); MrBPD = 29.77 (5.44); MHCs = 27.05 (7.17)	NI	MBPD = 24.91 (5.56), NI; MrBPD = 24.04 (6.60), NI; MHCs = 23.40 (5.32), NI	Cross-sectional	IPDE, BSL-23	SBA	Participants with rBPD: ↑ positive evaluation of their own body than cBPD who evaluated their own body significantly negatively	General body dissatisfaction: cBPD > rBPD and HCs in the negative evaluation of their own body; negative body image in the cBPD group but positive in the rBPD group in terms of neutral body areas. In cBPD and rBPD: ↑ negative evaluation of sexually connoted body areas than in HCs	Small sample size; only women; age limit (max. 50 years old); no mean education level
Kleindienst et al. [19]	Germany	BPD (N = 80; 80); PTSD (N = 36; 36); AD (N = 37; 37); HCs (N = 47; 47); wait-list patients	MBPD = 32.40 (9.68); MPTSD = 36.75 (9.24); MAD = 35.84 (11.72); MHCs = 31.23 (11.52); NI for each group separately, but 18–59 for the whole sample	NI	MBPD = 28.74 (8.62), NI; MPTSD = 26.33 (8.70), NI; MAD = 25.63 (10.23), NI; MHCs = 24.74 (5.65), NI	Cross-sectional	IPDE, BSL-23	SBA	BPD: ↑ negative self-evaluation with and without PTSD or reported CSA and in other groups; BPD + CSA and BPD–CSA had similar scores in negative body rati ng	General body dissatisfaction: mean evaluation of body areas differed across the 4 diagnostic groups; HCs > PTSD > BPD in average body rating	Only self-reported measures of body image; no mean education level; only women; small sample size (HCs); a speculative nature of scars
Muehlenkamp et al. [23]	USA	N = 398; 297; undergraduate students	M = 20.25 (2.45), NI	NI	NI, NI	Cross-sectional	BEST	BAS (4 subscales)	Negative body image as a moderator of the relation between difficulties in emotion regulation and NSSI	Perception: negative associations in body regard and NA, emotional dysregulation. and BPD symptoms; a positive association between NSSI and emotional dysregulation, BPD symptoms and NA. NSSI frequency significantly associated with emotional dysregulation and low body regard	Only self-reported measures of BPD and body image; only a nonclinical sample; no mean education level; no mean BMI
Sansone et al. [24]	USA	N = 48; 48 outpatients	M = 32.98 (9.28), 18–56	NI, but 85.4% women graduated from high school and 22.9% earned an academic degree	NI, but 17 women had BMI > 27.3 (obesity cut-off), NI	Cross-sectional	PDQ-R (BPD subscale)	BIAQ, EDI (BD subscale), 2 items on attractiveness	Patients with BPD features reported ↓ general body satisfaction, ↓ attractiveness, ↓ facial attractiveness, ↑ social avoidance due to body image concerns	Perception: PDQ-R scores positively corelated with BMI and other body image measures; obese women > nonobese women in PDQ-R. General body dissatisfaction: scores on PDQ-R positively correlated with body dissatisfaction.	Only self-reported measures of BPD and body image; no HCs group; no mean education level; no mean BMI; only women
Sansone et al. [2]	USA	N = 126; 126; inpatients	M = 34.84 (12.19), 18–74	NI, but 15.1% did not graduate from high school, 24.4% earned at least a 4-year college degree, and 5.9% earned a graduate degree	NI, NI	Cross-sectional	PDQ-4 (BPD subscale)	ASI (BIVS subscale)	Patients with BPD features reported ↑ negative evaluation of appearance	Affect/Cognition: ↑ BPD features => body image vulnerability, appearance stereotyping, private self-consciousness, public self-consciousness, and a lack of familiarity with one’s own body.	Only self-reporedt measures of BPD and body image; no mean education level; no mean BMI; only women
Steiger et al. [25]	Canada	BPD (N = 49; 49); BD/BPD (N = 22; 22); BD (N = 38; 38); BN (N = 22; 22); LR (N = 418; 418); high-school students (BPD, BD/BPD; BD, LR) and inpatients (BN)	MBPD = 15.47 (2.26); MBD/BPD = 16.59 (4.85); MBD = 15.53 (1.80); MBN = 24.50 (4.17); MLR = 15.23 (2.16); NI for the BN group, but 12–18 for the remaining 4 groups	NI	MBPD = 20.61 (2.70), NI; MBD/BPD = 21.45 (2.22), NI; MBN = 22.45 (2.77), NI; MBD = 22.53 (2.44), NI; MLR = 20.50 (2.09), NI	Cross-sectional	BSI	EDI (BDS subscale)	Girls with high body dissatisfaction and severe BPD features showed ↑ eating disorders symptoms, but ↓ than women with BN	General body dissatisfaction: BD/BPD did not significantly differ from BN in the degree of body dissatisfaction	Only self-reported measures of BPD and body image; no mean education level; only women; significant age differences between patients with BN and all other groups; no control for mean BMI; small sample sizes in the BD/BPD and BN groups
Witthöft et al. [21]	Germany	BPD + PTSD (N = 29; 29); PTSD (N = 32; 32); HCs (N = 30; 30); inpatients	MBPD + PTSD = 30.72 (8.62), NI; MPTSD = 39.28 (10.15), NI; MHCs = 32.80 (12.01), NI	NI, but ≥10 years was the case for: 73.1% of BPD + PTSD; 58.1% of PTSD; 96.4% of HCs	MBPD + PTSD = 27.69 (7.17), NI; MPTSD = 28.38 (7.49), NI; MHCs = 25.37 (5.52), NI	Experi-mental	IPDE, BSL-23	EST	Patients with BPD + PTSD reported ↑ attentional bias toward body-related stimuli than those with PTSD and HCs	Affect/cognition : PTSD + BPD > HCs in bias on words related to the body	No self-reported measure of body image; indirect measure of body image; no group with BPD without comorbid PTSD; significant age and education level differences between some of the groups; no mean education level; only women

Groups: A/S—scars after an accident/surgery; BD—body dissatisfaction; BD/BPD—body dissatisfaction and BPD traits; BN—bulimia nervosa; BPD—borderline personality disorder; CSA, child sexual abuse; HCs—healthy controls; NSSI—nonsuicidal self-injury; LR—low risk; PTSD—post-traumatic stress disorder; NA—negative affect; BID—body image disturbances, LDED—life diagnosis eating disorders; BPD measures: BEST—Borderline Evaluation of Severity over Time; BPI—Borderline Personality Inventory; BSI—Borderline Syndrome Index; BSL-23—Borderline Symptom List 23; IPDE—International Personality Disorder Examination; PDQ-4—Personality Diagnostic Questionnaire 4; PDQ-R—Personality Diagnostic Questionnaire—Revised, EDE-Q—Eating Disorder Examination Questionnaire. Body image measures: ASI (BIVS subscale)—Appearance Schemas Inventory (Body Image Vulnerability Scale); BAS—Body Attitudes Scale; BIAQ—Body Image Avoidance Questionnaire; EDI (BD subscale)—Eating Disorder Inventory (Body Dissatisfaction subscale); EST—Emotional Stroop Test; FKBB (EBIABI)—Evaluation of Body Image After Burn Injuries; MBSRQ-AS—Multidimensional Body–Self Relations Questionnaire—Appearance Scales; modified SBA—modified Survey of Body Areas, BIAQ—Body Image Avoidance Questionnaire. Other acronyms: NI—no information. Symbols: ↑—high; ↓—low.

**Table 2:** Characteristics of the measures used in the reviewed studies (for a review on body image measures, see [26]).

Body Image Measure	Reference	Components of Body Image Measured in the Reviewed Studies
ASI (BIVS subscale)	Cash, Labarge [24]	Affect and cognition
BAS	Walsh [22]	Body perception (body regard)
BIAQ	Rosen et al. [23]	Body perception
EDI (BDS subscale)	Garner et al. [25]	General body dissatisfaction
FKBB (EBIABI)	Seehausen et al. [27]	Body perception (originally designed to specifically measure body perception with regard to burn injuries)
MBSRQ-AS	Brown et al. [28]; Cash [29]	Affect and cognition
Modified SBA	Dyer et al. [14]; original version: Kleindienst et al. [15]	General body dissatisfaction

Body image measures: ASI (BIVS subscale)—Appearance Schemas Inventory (Body Image Vulnerability Scale); BAS—Body Attitude Scale; BIAQ—Body Image Avoidance Questionnaire; EDI (BD subscale)—Eating Disorder Inventory (Body Dissatisfaction subscale); FKBB (EBIABI)—Evaluation of Body Image After Burn Injuries; MBSRQ-AS—Multidimensional Body–Self Relations Questionnaire—Appearance Scales; modified SBA—modified Survey of Body Areas. We did not include the Emotional Stroop Test (EST) in the above measures, as it is an indirect measure of body image and therefore does not concern any of its specific components.
